# Supplementary material for: Estimating the time of Highly Pathogenic Avian Influenza virus introduction into United States poultry flocks during the 2022/24 epizootic
Source: PLoS One. 2024 Dec 13;19(12):e0310733. doi: 10.1371/journal.pone.0310733 (PMC11642945; doi:10.1371/journal.pone.0310733)
Supplement: Attachment — Submitted filename: PONE-D-24-38916_Comments to the author.docx [file pone.0310733.s001.docx]

**Estimating adequate contact rates and time of Highly Pathogenic Avian Influenza virus introduction into individual United States commercial poultry flocks during the 2022/24 epizootic (PONE-D-24-38916)**

The manuscript presents an important analysis of the introduction time (TOI) and adequate contact rate (ACR) for HPAI outbreaks in layer, broiler, and turkey flocks in the US during the 2022/24 epizootic. The authors employ mathematical modeling techniques, including the Approximate Bayesian Computation (ABC) algorithm alongside a stochastic within-flock HPAI transmission model, to estimate the detection time of HPAI in flocks. While the study offers valuable insights, I have some suggestions and questions that need to be addressed to improve the manuscript.

1. **General Comments:**

The manuscript utilizes complex sentence structures that may impede reader comprehension. Simplifying these sentences will enhance clarity and improve understanding of the results. Additionally, several grammatical errors throughout the text should be rectified to elevate the manuscript's overall quality.

2. **Specific revisions:**

Lines 38-40: Please revise for clarity and remove the extraneous bracket.

Line 61: Use the numeral '14' instead of spelling out 'fourteen.'

Line 71: This citation appears unnecessary and should be removed.

Lines 74-77: The content is unclear and lacks coherence; please revise for better flow.

Lines 105-106: This sentence appears incomplete; please clarify.

Line 114: Did you mean "Individual flock-based"? If so, please revise for accuracy.

Lines 204-205: There seems to be an error in the citation; please check.

Line 281: Remove the unnecessary comma.

Line 321: Use either "However" or "Most importantly," but not both

Authors Contribution section should be added

3. **Methods - Data:** The 'Data' section does not provide sufficient detail regarding the data utilized in this study. It is essential to include the following information:

The total number of farms/flocks that were infected.

The distribution of farms by species, rearing system, and other relevant parameters.

The inclusion and exclusion criteria applied to the flock data.

Providing this information will enhance the transparency and reproducibility of your findings

4. **Results:** The results section could benefit from clearer organization. Consider dividing the results into specific sub-sections or sub-headings to facilitate better understanding.
5. **Specific Questions:**
1) Did your analyses include any back-calculation methods? If so, please elaborate on how these were integrated into your study.
2) When comparing your results to other studies, particularly the Dutch 2003 epidemic, how do you account for differences in data, including strain type, geographical region, and variations in self-reporting or testing days?

Addressing these points will strengthen the manuscript and provide clearer insights into your research. Thank You!
