# Supplementary material for: Estimating the time of Highly Pathogenic Avian Influenza virus introduction into United States poultry flocks during the 2022/24 epizootic
Source: PLoS One. 2024 Dec 13;19(12):e0310733. doi: 10.1371/journal.pone.0310733 (PMC11642945; doi:10.1371/journal.pone.0310733)
Supplement: Attachment — Submitted filename: Response to Reviewers.docx [file pone.0310733.s002.docx]

When submitting your revision, we need you to address these additional requirements.

1. Please ensure that your manuscript meets PLOS ONE's style requirements, including those for file naming. The PLOS ONE style templates can be found at

https://journals.plos.org/plosone/s/file?id=wjVg/PLOSOne_formatting_sample_main_body.pdf and

<https://journals.plos.org/plosone/s/file?id=ba62/PLOSOne_formatting_sample_title_authors_affiliations.pdf>

***The files have been renamed as per the guidelines.***

2. Please note that PLOS ONE has spec6ific guidelines on code sharing for submissions in which author-generated code underpins the findings in the manuscript. In these cases, all author-generated code must be made available without restrictions upon publication of the work. Please review our guidelines at https://journals.plos.org/plosone/s/materials-and-software-sharing#loc-sharing-code and ensure that your code is shared in a way that follows best practice and facilitates reproducibility and reuse.

***The author-generated code will be readily without restrictions.***

3. Thank you for stating the following financial disclosure:

“This study was funded through a cooperative agreement between the Center for Epidemiology and Animal Health (CEAH) of the USDA, Animal and Plant Health Inspection Service (APHIS) Veterinary Services (VS) and the University of Minnesota (UMN) as USDA Award # AP22VSSP0000C051 (Quantitative Analysis and Support for Agricultural Animal Disease Emergency Preparedness and Response in the U.S.). The findings and conclusions in this document are those of the authors and should not be construed to represent any official USDA or U.S. Government determination or policy.”

Please state what role the funders took in the study.  If the funders had no role, please state: "The funders had no role in study design, data collection and analysis, decision to publish, or preparation of the manuscript."

If this statement is not correct you must amend it as needed.

Please include this amended Role of Funder statement in your cover letter; we will change the online submission form on your behalf.

***We have amended the financial disclosure to include the statement “…USDA co-authors assisted with data collection and preparation of the manuscript as part of work performed under the cooperative agreement. At the time of manuscript preparation, the cooperative agreement was not directly managed by any of the co-authors.…” in lines 393-396.***

4. We note that you have indicated that there are restrictions to data sharing for this study. PLOS only allows data to be available upon request if there are legal or ethical restrictions on sharing data publicly. For more information on unacceptable data access restrictions, please see http://journals.plos.org/plosone/s/data-availability#loc-unacceptable-data-access-restrictions.

Before we proceed with your manuscript, please address the following prompts:

a) If there are ethical or legal restrictions on sharing a de-identified data set, please explain them in detail (e.g., data contain potentially identifying or sensitive patient information, data are owned by a third-party organization, etc.) and who has imposed them (e.g., a Research Ethics Committee or Institutional Review Board, etc.). Please also provide contact information for a data access committee, ethics committee, or other institutional body to which data requests may be sent.

***The data used as part of this study were collected by USDA as part of disease outbreak response activity purposes and are considered confidential business information. Anonymized data may be made available upon request, for statistical purposes only. Requests to access the datasets should be directed to HCH.***

b) If there are no restrictions, please upload the minimal anonymized data set necessary to replicate your study findings to a stable, public repository and provide us with the relevant URLs, DOIs, or accession numbers. For a list of recommended repositories, please see

https://journals.plos.org/plosone/s/recommended-repositories. You also have the option of uploading the data as Supporting Information files, but we would recommend depositing data directly to a data repository if possible.

We will update your Data Availability statement on your behalf to reflect the information you provide.

Review Comments to the Author

Reviewer #1: The manuscript presents an important analysis of the introduction time (TOI) and adequate contact rate (ACR) for HPAI outbreaks in layer, broiler, and turkey flocks in the US during the 2022/24 epizootic. The authors employ mathematical modeling techniques, including the Approximate Bayesian Computation (ABC) algorithm alongside a stochastic within-flock HPAI transmission model, to estimate the detection time of HPAI in flocks. While the study offers valuable insights, I have some suggestions and questions that need to be addressed to improve the manuscript (detailed in the attached file).

**Estimating adequate contact rates and time of Highly Pathogenic Avian Influenza virus introduction into individual United States commercial poultry flocks during the 2022/24 epizootic (PONE-D-24-38916)**

The manuscript presents an important analysis of the introduction time (TOI) and adequate contact rate (ACR) for HPAI outbreaks in layer, broiler, and turkey flocks in the US during the 2022/24 epizootic. The authors employ mathematical modeling techniques, including the Approximate Bayesian Computation (ABC) algorithm alongside a stochastic within-flock HPAI transmission model, to estimate the detection time of HPAI in flocks. While the study offers valuable insights, I have some suggestions and questions that need to be addressed to improve the manuscript.

***Thank you for the compliments and the subsequent comments as they will help us improve the manuscript.***

1. **General Comments:**

The manuscript utilizes complex sentence structures that may impede reader comprehension. Simplifying these sentences will enhance clarity and improve understanding of the results. Additionally, several grammatical errors throughout the text should be rectified to elevate the manuscript's overall quality.

***This has been addressed through several changes as highlighted in track changes version of the manuscript. Also, the effected changes are captured in the individual rebuttals below.***

2. **Specific revisions:**

Lines 38-40: Please revise for clarity and remove the extraneous bracket.

***This has been addressed directly and also as part of the corrections suggested by the other reviewer in line 42-47 as follows: “…The 2022/24 H5N1 Highly Pathogenic Avian Influenza (HPAI) clade 2.3.4.4b outbreak in North American poultry and other species was preceded by detections in wild birds. The initial detection was from a deceased Great Black-backed Gull submitted to the Canadian Wildlife Health Cooperative in late 2021 (Giacinti et al., 2024); subsequently, the first detections in the United States occurred in January 2022 and were among wild ducks sampled for USDA active wild bird surveillance (USDA APHIS, 2024d). …”.***

Line 61: Use the numeral '14' instead of spelling out 'fourteen.'

***This has been changed accordingly in line 68.***

Line 71: This citation appears unnecessary and should be removed.

***The citation of (Center for Epidemiology and Animal Health, 2023) has been removed from line 78 as suggested.***

Lines 74-77: The content is unclear and lacks coherence; please revise for better flow.

***The content has been revised for improved clarity and coherence in lines 81-86 as “…To elucidate the possible discrepancies between the two outbreaks, the USDA performed case-control studies for commercial turkey (Patyk et al., 2023) and commercial table egg layer flocks (Green et al., 2023). Collectively from the two studies, the identified risk factors for HPAIV introduction included being in a disease control zone, increased sightings of wild waterfowl in the vicinity of the poultry premises, and offsite rendering of farm byproducts, among others….”***

Lines 105-106: This sentence appears incomplete; please clarify.

***This has been revised for improved clarity in lines 114-116 as “…The data used in this analysis included daily mortality records and diagnostic laboratory submission reports and test results….”***

Line 114: Did you mean "Individual flock-based"? If so, please revise for accuracy.

***The transmission model tracks every bird in the flock individually through the different disease stages that it undergoes. Such models are broadly referred to as “individual-based models”. However, in order to eliminate the ambiguity about the epidemiological unit of interest, we have adopted the term “individual bird-based model” and we have revised the sentence in lines 116-118 as “…A stochastic SEIR (i.e., susceptible, latently infected, infectious, and removed (dead)) individual bird-based HPAI transmission model was used to simulate the disease induced mortality and infection prevalence. …”***

Lines 204-205: There seems to be an error in the citation; please check.

***This error regrettably arose from the creation of the pdf version by the submission system. The original lines 233-235 read as follows “…For the reported index commercial poultry case of the current outbreak in the U.S., the observed and model predicted outcomes for mortality data are presented in Figure 1…”.***

Line 281: Remove the unnecessary comma.

***The extra comma has been removed.***

Line 321: Use either "However" or "Most importantly," but not both

***In line 351, we have changed the phrasing to use “Notwithstanding” and the sentence now reads “…Notwithstanding, including these flocks in the analysis did not affect the trend observed in the species-specific comparison of outcomes….”***

Authors Contribution section should be added

***We have added the following to this effect in lines 403-408:***

***“…Author Contributions***

***A.S. and S.M. performed the analyses and A.S. drafted the article. A.S., S.M., P.J.B., K.M.S., H.C.H., M.S., R.M., M.R.C., and C.J.C. conceived the analysis. H.C.H and M.S. acquired the data in coordination with field epidemiologists and response personnel. S.M., P.J.B., K.M.S., H.C.H., M.S., R.M., M.R.C., and C.J.C. provided substantive revisions to the article. All authors reviewed the manuscript and approved the final version submitted….”***

3. **Methods - Data:** The 'Data' section does not provide sufficient detail regarding the data utilized in this study. It is essential to include the following information:

The total number of farms/flocks that were infected.

The distribution of farms by species, rearing system, and other relevant parameters.

The inclusion and exclusion criteria applied to the flock data.

Providing this information will enhance the transparency and reproducibility of your findings

***This has been addressed through the following changes:***

***1) In data section, lines 114-128 as “…The data used in this analysis included daily mortality records and diagnostic laboratory submission reports and test results. Mortality records are provided to the USDA for indemnity purposes; laboratory documents for positive premises are recorded in USDA’s Emergency Management Response System. This data was deidentified and provided to the Secure Food Systems (SFS) Team at the University of Minnesota for analysis via a material transfer agreement. Flock data provided to the SFS team reflect a convenience sample of infected premises. Infected premises which were the first detection in a State and/or thought to be involved in cases of lateral transmission were selected for analysis to support ongoing field investigations. Furthermore, premises with inadequate data (e.g., mortality records provided in aggregate instead of daily, unknown sampling schemes) or that had abnormal baseline mortality patterns, likely due to underlying comorbidities (e.g., avian metapneumovirus), were excluded. Commercial premises infected between February 2022 and December 31, 2023 were eligible for inclusion. At this time, 452 commercial premises infected with HPAIV were confirmed detected. Detections included 39 commercial broiler premises, 55 commercial table egg layer premises, and 308 commercial turkey premises…”***

***2) In results’ section, lines 230-233 as “…The subset of premises analyzed reflect 18% (72 out of 402) of all infected broiler (9 out of 39), layer (18 out of 55), and turkey (45 out of 308) premises during the data period; split by epidemic phase, 24% (42 out of 174) of premises during end -July’22 and 13% (30 out of 228) of premises post-July’22 were analyzed….”***

4. **Results:** The results section could benefit from clearer organization. Consider dividing the results into specific sub-sections or sub-headings to facilitate better understanding.

***We have made the following changes to this effect: We have changed the very first paragraph in results in lines to be read “…The subset of premises analyzed reflect 18% (72 out of 402) of all infected broiler (9 out of 39), layer (18 out of 55), and turkey (45 out of 308) premises during the data period; split by epidemic phase, 24% (42 out of 174) of premises during end -July’22 and 13% (30 out of 228) of premises post-July’22 were analyzed…”. Then the second paragraph introduces the example graph on the comparison between the model output and field derived mortality data, the next paragraphs introduce results in Table 2 which presents an overall summary of the number of test days and the results on the estimated MTFPS, within-flock ACR, and*** $\boldsymbol{R}_{\boldsymbol{0}}$***. After presenting the overall results, we then zoom in on the species-specific results followed by the epidemic-phase specific results. Lastly we present species-specific results grouped by the ranking of the adequate contact rate into slow, moderate and fast spread.***

5. **Specific Questions:**
1) Did your analyses include any back-calculation methods? If so, please elaborate on how these were integrated into your study.

***The study did not use a back-calculation method. It used a “forward simulation approach” in which the model predicted outcomes for a given set of parameters drawn from the given prior distributions were compared with the observed field data as described in the methods. Using a distance measure, the set of parameter values meeting the inclusion criteria were selected and summarized as outputs of interest. Note that this forward simulation approach enables considering the variability in disease durations among individual birds more directly and is beneficial to jointly estimate the adequate contact rate and the time of virus introduction***.

2) When comparing your results to other studies, particularly the Dutch 2003 epidemic, how do you account for differences in data, including strain type, geographical region, and variations in self-reporting or testing days?

***We address this by emphasizing caution when attempting to compare results involving different virus strains, different farm management practices as is the case between Europe and the U.S. We only mention the highlighted studies as being “closely related” e.g. in terms of methods and/or the data used as well as species and generic HPAI disease. To this effect, we stated in lines 308-315 that “…However, comparing findings from studies involving different virus strains should be done with caution. Note that HPAI virus strain-specific characteristics (Spickler et al., 2008), production type, and flock management practices may influence within flock disease spread dynamics. As examples of closely related studies using field HPAI outbreak mortality data in commercial turkey flocks to estimate disease parameters, an ACR of 3.2 (95% CI: 2.3, 4.3) per day and*** $\boldsymbol{R}_{\boldsymbol{0}}$ ***of 12.8 (95% CI: 9.2, 17.2) were estimated for the 2014/15 U.S. H5N2 epidemic (Ssematimba et al., 2019) and, an ACR of 3.37 (95% CI: 0.97, 11.74) per day was estimated for the Dutch 2003 H7N7 epidemic (Bos et al., 2009)….”***

Addressing these points will strengthen the manuscript and provide clearer insights into your research. Thank You!

***Indeed these comments have been very well received and adjustments to the manuscript have been made. We greatly appreciate the efforts dispensed.***

Reviewer #2: Title need to be short specific and simplified.

***The title has be shortened to: “******Estimating the time of Highly Pathogenic Avian Influenza virus introduction into United States poultry flocks during the 2022/24 epizootic”***

From line 18-23, 26-28, need to be modified.

***For improved content flow and clarity, we split the sentences and rephrased the content in lines 18-27 as follows: “…In this study, we used changes in mortality rates as a proxy for HPAI incidence in a layer, broiler and turkey flock together with diagnostic test results to infer within-flock HPAI transmission dynamics. Mathematical modeling techniques, specifically the Approximate Bayesian Computation algorithm in conjunction with a stochastic within-flock HPAI transmission model were used in the analysis. The time window of HPAI virus introduction into the flock (TOI) and the adequate contact rate (ACR) were estimated. Then, using the estimated TOI together with the day when the first HPAI positive sample was collected from the flock, we calculated the most likely time to first positive sample (MTFPS) which reflects the time to HPAI detection….”***

Line 30 – related studies.

***We have improved clarity of this sentence in lines 30-34 by first splitting it into two and rephrasing it to: “…We conclude that this findings from this and other related studies are beneficial for the different stakeholders in outbreak management. We recommend that combining TOI analysis with complementary approaches such as phylogenetic analyses is critically important for improved understanding of disease transmission pathways...”***

Line 33-34 The estimated parameters can also inform models used for surveillance design, risk analysis, and emergency preparedness.

***To improve clarity and content flow, we have rephrased lines 35-38 as follows: “…The estimated parameters can also be used to parametrize mathematical models that can guide the design of surveillance protocols, risk analyses of HPAI spread, and emergency preparedness for HPAI outbreaks….”***

Line 39 was preceded by detections in wild birds. What type of wild birds and the sentence need to be reformed. Citation not provided.

***This has been addressed in lines 42-47 that now reads as follows: “…The 2022/24 H5N1 Highly Pathogenic Avian Influenza (HPAI) clade 2.3.4.4b outbreak in North American poultry and other species was preceded by detections in wild birds. The initial detection was from a deceased Great Black-backed Gull submitted to the Canadian Wildlife Health Cooperative in late 2021 (Giacinti et al., 2024); subsequently, the first detections in the United States occurred in January 2022 and were among wild ducks sampled for USDA active wild bird surveillance (USDA APHIS, 2024d). …”.***

Line 40-41 writing error please check.

***This error has been catered for through the changes reflected in lines 42-47 that now read as follows: “…The 2022/24 H5N1 Highly Pathogenic Avian Influenza (HPAI) clade 2.3.4.4b outbreak in North American poultry and other species was preceded by detections in wild birds. The initial detection was from a deceased Great Black-backed Gull submitted to the Canadian Wildlife Health Cooperative in late 2021 (Giacinti et al., 2024); subsequently, the first detections in the United States occurred in January 2022 and were among wild ducks sampled for USDA active wild bird surveillance (USDA APHIS, 2024d). …”.***

Line 140 please provide Citation.

***We have addressed this introducing the following in lines 164-166: “…Similar distance measures based on squared difference between the observed and model predicted mortality have been utilized in other outbreak analyses (Guinat et al., 2018; Nezworski et al., 2021)…” and in lines 170-171 “…The matching of observed and simulated test results in the acceptance criteria of the ABC algorithms was also prioritized in Nezworski et al. (2021)….”***

Line 176-177 author mention about graph but didn’t mention any graph no in the manuscript. Citation required.

***This was an error related to the system error in generating the pdf version of the manuscript were the citation did not typeset correctly. The graph was generated for this study (hence no citation is required) and it is presented as Figure 1 in the results’ section. It is talked about in lines 210-213 as follows: “…For the reported index commercial poultry case of the current outbreak in the U.S., the observed and model predicted outcomes for mortality data are presented in Figure 1. The estimated daily mortality from the model closely match the field mortality data for this flock, indicating a reasonable fit….”. The figure is uploaded as a separated file and as per the journal guidelines, its caption is located towards the end of the manuscript in lines 527-528 and reads as follows: “…Figure 1. Observed (red triangles) and model predicted daily mortality (grey band) for the index commercial poultry case of the 2022/24 U.S. H5N1 HPAI epidemic…”***

Line 180-184 author please clarify the sentence and need to be rewrite.

***These sentences were intended to explain the criteria for defining the two epidemic phases but lacked clarity as were written. They have been split and rewritten in lines 205-211 as follows: “…Note that there were no new reported HPAI cases in commercial broilers, layers, and turkeys from July 26 to August 22, 2022 (USDA APHIS, 2024a). Therefore, in this analysis the epidemic phase cutoff was set to July 31, 2022 and of the then 402 reported outbreaks in commercial broilers, egg layers, and turkeys, 174 outbreaks occurred prior to July 26, 2022 and 228 occurred between August 22, 2022 and December 31, 2023. …”***

Line 204-205 citation error.

***As also spotted by reviewer #1 above,*** ***this error regrettably arose from the creation of the pdf version by the journal submission system. The original lines 204-205 read as follows “…For the reported index commercial poultry case of the current outbreak in the U.S., the observed and model predicted outcomes for mortality data are presented in Figure 1. .”***

In methos analysis line 177-184 data does not match with the result showing in line no 207-210. Please clarify this.

***The total number of reported cases was 452 and 402 of these were in commercial broilers, egg layers, and turkeys. However, of the 402 cases in commercial poultry flocks, only 72 (i.e., 9 broilers, 18 layers and 45 turkeys) met inclusion criteria as now described in lines 120-129 that now reads “… Flock data provided to the SFS team reflect a convenience sample of infected premises. Infected premises which were the first detection in a State and/or thought to be involved in cases of lateral transmission were selected for analysis to support ongoing field investigations. Furthermore, premises with inadequate data (e.g., mortality records provided in aggregate instead of daily, unknown sampling schemes) or that had abnormal baseline mortality patterns, likely due to underlying comorbidities (e.g., avian metapneumovirus), were excluded. Commercial premises infected between February 2022 and December 31, 2023 were eligible for inclusion. At this time, 452 commercial premises infected with HPAIV were confirmed detected. Detections included 39 commercial broiler premises, 55 commercial table egg layer premises, and 308 commercial turkey premises.”. We have also introduced summary results in the results section in lines 230-233 that reads “…The subset of premises analyzed reflect 18% (72 out of 402) of all infected broiler (9 out of 39), layer (18 out of 55), and turkey (45 out of 308) premises during the data period; split by epidemic phase, 24% (42 out of 174) of premises during end -July’22 and 13% (30 out of 228) of premises post-July’22 were analyzed….”***

In Discussion language very confusing please simplify the sentences to highlight your outcomes with the previously available database.

***Language in and sections of the discussion, specifically throughout lines 292 through 393, have been modified to provide improved clarity. The now improved lines 292 to 313 read as follows “…When all flocks were grouped by phase without consideration for species, there was only a slight decrease from a median ACR of 6.8 to 6.6 birds per day and in*** $\boldsymbol{R}_{\boldsymbol{0}}$ ***from 15 to 13 birds when observing differences between the first phase, End-July’22, and the second phase, Post-July’22. However, the observed decreased between phases was not significant? When grouped by both species and epidemic phase there were no significant differences observed. The negligible change in these estimates based on phase may be indicative of the fact that the virus did not change much in its transmissibility characteristics across the two phases.***

***By species alone, the estimated median ACR and*** $\boldsymbol{R}_{\boldsymbol{0}}$***were highest in turkey flocks and lowest in broiler flocks. Moreover, turkey flocks were observed to have significantly higher*** $\boldsymbol{R}_{\boldsymbol{0}}$ ***in comparison to broiler flocks. The differences in estimated median ACR and*** $\boldsymbol{R}_{\boldsymbol{0}}$ ***indicate that the virus was significantly more transmissible in turkeys than in broilers and layers (Table 2). Higher transmissibility of HPAI virus between turkeys in comparison to between chickens has also been reported in previous studies (Tumpey et al., 2004; Puranik et al., 2020). Additionally, findings in the present study are in agreement with the outcomes from experimental studies by Pantin-Jackwood et al. (2023) in which the authors demonstrated that successful infection of chickens required a higher inoculation dose compared to turkeys, and chickens had shorter mean death times than turkeys. Similar observations were made in the study by Puranik et al. (2020) wherein the observed extended mean time to death in turkeys results in a longer virus shedding period and hence enhances transmission to contact birds (i.e., higher*** $\boldsymbol{R}_{\boldsymbol{0}}$***).…”***

***Lines 337 through 344 now read as follows “…Overall, these results suggest that the most likely time to first positive sample and detection was relatively long for commercial broiler flocks (7.0 [6.0, 9.0] days) compared to other species and the number of test days were few (2.0 [1.0, 2.0] days). The longer time to detect paired with a smaller number of test days is an indicator of a likely negative correlation between the two measures. In contrast, the results were the exact opposite in egg layers (MTFPS: 4.0 [3.0, 5.8] days; Median test days: 3.0 [1.3, 4.8] days). Possible explanations for this apparently “unbalanced” delay in HPAI detection and frequency of diagnostic testing may partly lie in each species’ reasons for testing…”***

***Lines 349 through 355 now read as follows “…The diagnostic testing related to egg movements typically occurs at higher frequency than the testing required for single-use permits related to terminal or transfer live bird movements that are common in turkey or broiler production. The more frequent testing required by for egg laying flocks that enables egg movements off farms may explain the higher number of test days, and hence the relatively shorter MTFPS, in layers compared to other analyzed species that are focused on live bird movements….”***

***Lines 360 through 367 now read as follows “…Given that the average market age of commercial broilers is 47 days (National Chicken Council, 2023), the window for required testing is smaller for broiler flocks compared to market ages of grow-out turkeys (i.e., 98 to 140 days)(National Turkey Federation, 2024). Additionally, the majority of the broilers in the U.S. are raised in the southeastern part of the country where there were many fewer HPAI cases and thus, many fewer control areas. Therefore, susceptible broiler farms that ended up becoming infected may not have been as likely to end up in preexisting control areas, and were thus not as often subject to control area-mandated surveillance testing in the first place…”***

***Additionally, we believe that results have been compared with sufficient literature within the field. Examples on such include the content in edited lines 304 through 311 as follows “…Higher transmissibility of HPAI virus between turkeys in comparison to between chickens has also been reported in previous studies (Tumpey et al., 2004; Puranik et al., 2020). Additionally, findings in the present study are in agreement with the outcomes from experimental studies by Pantin-Jackwood et al. (2023) in which the authors demonstrated that successful infection of chickens required a higher inoculation dose compared to turkeys, and chickens had shorter mean death times than turkeys. Similar observations were made in the study by Puranik et al. (2020) wherein the observed extended mean time to death in turkeys results in a longer virus shedding period and hence enhances transmission to contact birds (i.e., higher*** $\boldsymbol{R}_{\boldsymbol{0}}$***)…” and in lines 330 through 336 as follows “…As examples of closely related studies using field HPAI outbreak mortality data in commercial turkey flocks to estimate disease parameters, an ACR of 3.2 (95% CI: 2.3, 4.3) per day and*** $\boldsymbol{R}_{\boldsymbol{0}}$ ***of 12.8 (95% CI: 9.2, 17.2) were estimated for the 2014/15 U.S. H5N2 epidemic (Ssematimba et al., 2019) and, an ACR of 3.37 (95% CI: 0.97, 11.74) per day was estimated for the Dutch 2003 H7N7 epidemic (Bos et al., 2009). Generally, when comparing the two large U.S. HPAI epidemics, the 2022/24 outbreak virus strain was more infectious than that of 2014/15 outbreak virus strain (Pantin-Jackwood et al., 2023)…”. Much of other topics of discussion (lines 337 and beyond) have limited peer-reviewed literature to compare to, thus general industry knowledge is referenced as part of the discussion.***

References are not properly cited in the main text.

***This error regrettably occurred in lines 204-205 when the system attempted to capture the reference to Figure 1 as a citation and this should be rectified.***
